# The Resin from *Protium heptaphyllum* Prevents High-Fat Diet-Induced Obesity in Mice: Scientific Evidence and Potential Mechanisms

**DOI:** 10.1155/2015/106157

**Published:** 2015-01-29

**Authors:** Karine Maria Martins Bezerra Carvalho, José Delano Barreto Marinho Filho, Tiago Sousa de Melo, Ana Jérsia Araújo, Josiane da Silva Quetz, Maria do Perpétuo Socorro Saldanha da Cunha, Karina Moura de Melo, Armenio Andre de Carvalho Almeida da Silva, Adriana Rocha Tomé, Alexandre Havt, Said Gonçalves da Cruz Fonseca, Gerly Anne de Castro Brito, Mariana Helena Chaves, Vietla Satyanarayana Rao, Flávia Almeida Santos

**Affiliations:** ^1^Post-Graduate Programme in Medical Sciences, Faculty of Medicine, Federal University of Ceará, 60430-140 Fortaleza, CE, Brazil; ^2^Department of Physiology and Pharmacology, Faculty of Medicine, Federal University of Ceará, 60430-270 Fortaleza, CE, Brazil; ^3^Faculty of Pharmacy, Odontology and Nurse, Federal University of Ceará, 60430-160 Fortaleza, CE, Brazil; ^4^INCT-IBISAB-Brazilian Semi-Arid Institute of Biomedicine, Faculty of Medicine, Federal University of Ceará, 60430-270 Fortaleza, CE, Brazil; ^5^Haroldo Juaçaba Hospital, Cancer Institute of Ceará, 60430-230 Fortaleza, CE, Brazil; ^6^Department of Organic Chemistry, Federal University of Piauí, 64049-550 Teresina, PI, Brazil; ^7^Faculty of Veterinary Medicine, State University of Ceará, 60740-000 Fortaleza, CE, Brazil; ^8^Department of Morphology, Faculty of Medicine, Federal University of Ceará, 60430-170 Fortaleza, CE, Brazil

## Abstract

Herbal compounds rich in triterpenes are well known to regulate glucose and lipid metabolism and to have beneficial effects on metabolic disorders. The present study investigated the antiobesity properties of resin from *Protium heptaphyllum* (RPH) and the possible mechanisms in mice fed a high-fat diet (HFD) for 15 weeks. Mice treated with RPH showed decreases in body weight, net energy intake, abdominal fat accumulation, plasma glucose, amylase, lipase, triglycerides, and total cholesterol relative to their respective controls, which were RPH unfed. Additionally, RPH treatment, while significantly elevating the plasma level of ghrelin hormone, decreased the levels of insulin, leptin, and resistin. Besides, HFD-induced increases in plasma levels of proinflammatory mediators TNF-*α*, IL-6, and MCP-1 were significantly lowered by RPH. Furthermore, *in vitro* studies revealed that RPH could significantly inhibit the lipid accumulation in 3T3-L1 adipocytes (measured by Oil-Red O staining) at concentrations up to 50 *μ*g/mL. These findings suggest that the antiobese potential of RPH is largely due to its modulatory effects on various hormonal and enzymatic secretions related to fat and carbohydrate metabolism and to the regulation of obesity-associated inflammation.

## 1. Introduction

Obesity has become a growing public health concern in the developed and developing world, since it increases the risk for diabetes type 2, cardiovascular and inflammatory diseases like steatohepatitis, and acute pancreatitis [1], which may have significant negative impacts on quality of life. The goal of all obesity therapies is negative energy balance, which is helpful to improve and even reverse obesity-related complications. To counter the rapid increase in adiposity, one approach is bariatric surgery, which can result in impressive weight loss and improvement of obesity-related comorbidities. However, anemia, which may affect as many as two-thirds of patients, is of concern [2]. Drugs have long been used, but numerous once-promising weight-loss drugs have been abandoned because of serious toxic effects. Following the withdrawal of rimonabant and sibutramine, the only FDA-approved medication for the long-term management of obesity was orlistat [3]. However, the clinical utility of this amylase and lipase inhibitor has been limited by its gastrointestinal side effects and marginal efficacy. Similarly, although the weight-loss efficacy of the noradrenergic drug combination phentermine/topiramate (Qsymia) has been demonstrated, its teratogenic potential and increased heart rate remain important concerns [4]. In recent years, the availability and popularity of natural dietary supplements for the treatment of obesity have risen dramatically, and many botanical species including crude extracts and isolated compounds from plants have been shown to provide potentially promising therapeutic effects, including appetite control and weight loss [5].

The oleoresins exuded from Amazonian species of the Burseraceae family, particularly* Protium heptaphyllum* (Aubl.) March, is rich in triterpenes that include the ursane-type triterpenes *α*-amyrin, *α*-amyrenone, and brein, and the oleanane-type triterpenes *β*-amyrin, *β*-amyrenone, and maniladiol [6–8]. In the past few years, the resin from* Protium heptaphyllum* and its major component, *α*,*β*-amyrin, have been reported to exhibit many bioactive properties such as antioxidant, anti-inflammatory, antimicrobial, antihyperglycemic, hypolipidemic, gastroprotective, and hepatoprotective properties [9–12]. Taking into account the reported antiobese potentials of ursolic and oleanolic acids [13, 14] and the well-established anti-inflammatory, antihyperglycemic, and hypolipidemic effects of *α*,*β*-amyrin [12], the major component of RPH that has close structural resemblance to ursane and oleanone series, prompted us to examine the antiobesity potential of RPH in mice fed a high-fat diet.

## 2. Materials and Methods

### 2.1. Plant Material

The resinous exudate from the trunk wood of* P. heptaphyllum* was collected from the municipal areas of Timon, Maranhão, Brazil, after its identification by botanist Roseli Farias de Melo Barros. A voucher sample (no. TEPB 18247) has been deposited at the Herbarium Graziela Barroso of the Federal University of Piauí. The crude resin (410 g) was dissolved in methanol/dichloromethane (4 : 1), filtered, and the solvent was evaporated in a Rotavapor to obtain 408 g (99.5%) of amorphous white resin. Phytochemical analysis on the resin revealed the presence of pentacyclic triterpenoids (56%), which was identified by ^1^H and ^13^C NMR and mass spectroscopy. These were the mixture of *α*- and *β*-amyrin (45.25%), brein and maniladiol (9.5%), and a small quantity (1.25%) of a mixture of lupenone and *α*- and *β*-amyrenone [15].

### 2.2. Animal and Experimental Protocol

Male Swiss mice weighing 25–30 g obtained from the Central Animal House of the Federal University of Ceará were used. They were housed in a controlled environment (24 ± 2°C, 55 ± 5% relative humidity, 12 h light/dark cycle) with food (chow) and water provided ad libitum unless otherwise noted. The Federal University of Ceará Institutional Committee on Care and Use of Animals approved experimental protocols (number 70/10) for experimentation, in accordance with the guidelines of the National Institutes of Health, Bethesda, MD.

The standardized high-fat diet used for the study [16] comprised the following hypercaloric constituents: 15 g of laboratory animal chow, 10 g of roasted ground nut, 10 g of milk chocolate, and 5 g of sweet biscuits. These ingredients were ground and prepared in the form of pellets containing 20% protein, 48% carbohydrate, 20% lipids, 4% cellulose, 5% vitamins and minerals, and 3% humidity by weight. The net energy content of this diet was 21.40 kJ/g. To avoid autoxidation of the fat components, food was stored at 2–8°C. Laboratory pellet chow was used as a control diet (Nuvilab, Colombo, PR, Brazil) that consisted of 19% protein, 56% carbohydrate, 3.5% lipids, 4.5% cellulose, 5.0% vitamins and minerals, and 12% humidity with a net energy content 17.03 kJ/g.

The animals were randomly divided into five groups (*n* = 8) matched for body weight after 1 week of being fed laboratory pellet chow. The control group continued to be fed laboratory pellet chow ad libitum and was designated ND (normal diet fed group). The remaining mice consumed a high-fat diet (HFD, control), HFD + resin of* P. heptaphyllum* (RPH, 0.05% in drinking water, which is equivalent to a dose of 10 mg/kg based on water consumption), HFD + resin of* P. heptaphyllum* (RPH, 0.1% in drinking water which is equivalent to 20 mg/kg), or HFD + sibutramine (SIB, 0.05% in drinking water, equivalent to 10 mg/kg) for 15 weeks. The choice for the drug concentrations adopted for RPH and SIB was based on preliminary studies that showed their safety and efficacy. RPH was suspended initially in 2% (v/v) Tween 80 and then further in water. HFD-fed controls received the same vehicle. Since SIB is water-soluble, no vehicle was used. RPH- or vehicle-water was changed twice a week, and weekly consumption of water (mL/week) was noted.

The body weight of each mouse was measured once a week, the total amount of food consumption was recorded every day for 15 weeks, and weekly consumption of food (g/week) and water (mL/week) was noted. Energy intake (kJ/mouse/day) was also evaluated during the 15 weeks of feeding trial. At the end of this period, animals were starved for 6 h, blood was taken by venous puncture under light anesthesia with diethyl ether, and then the mice were sacrificed by cervical dislocation. The plasma was used within a few hours or frozen at −70°C until analysis. The liver and abdominal adipose tissues (epididymal and parametrial) were dissected, weighed, and expressed in milligrams per 10 g body weight.

### 2.3. Biochemical Analysis

Plasma amylase and lipase were determined by a kinetic method using commercial kits for amylase (Labtest, Minas Gerais, Brazil) and lipase (Bioclin, Minas Gerais, Brazil). The assays were performed according to the manufacturer's instructions, and their levels were expressed in units per liter. Plasma glucose, triglycerides, and total cholesterol were analyzed using commercial kits (Labtest, Minas Gerais, Brazil), and the levels were expressed in milligrams per deciliter. Plasma alanine amino transferase (ALT) and aspartate amino transferase (AST) activities expressed in units per liter were analyzed by a kinetic method using commercial kits (Labtest, Minas Gerais, Brazil). The hepatic lipids were extracted using the Folch method [17], and triglycerides and total cholesterol concentrations were determined using commercial kits (Labtest, Minas Gerais, Brazil) and expressed in milligrams per gram (mg/g). Plasma insulin, leptin, ghrelin, resistin (Millipore, Billerica, MA, USA), TNF-*α*, IL-6, and MCP-1 (R&D Systems, Minneapolis, MN, USA) were measured by enzyme-linked immunosorbent assay (ELISA) performed in duplicate and expressed in nanograms or picograms per milliliter.

### 2.4. Quantitative Reverse Transcription-Polymerase Chain Reaction (qRT-PCR) Analysis

Total RNA was extracted from frozen adipose tissue (100 mg) using a QIAzol Lysis RNeasy Lipid Tissue Mini Kit (Qiagen, USA) according to the manufacturer's protocol. Equal amounts of total RNA were used for each analysis. The obtained RNA was reverse-transcribed with an iScript cDNA Synthesis Kit (Bio-Rad, USA) and quantified using NanoDrop 2000 (Thermo Scientific, USA). The synthesized cDNA served as a template in a 20 *μ*L reaction mixture. All primer sequences were determined through established GenBank sequences (NCBI number). Primer sequences were synthesized by Life Technologies (São Paulo, Brazil) as follows (5′–3′):* Mus musculus* peptidylprolyl isomerase A (PpiA): AAT­GCT­GGA­CCA­AAC­ACAAA (forward) and TTC­CAC­AAT­GTT­CAT­GCC­TT (reverse) (NM_008907.1);* Mus musculus* lipoprotein lipase (LPL): F-CCA­ATG­GAG­GCA­CTT­TCCA (forward) and CAC­GTC­TCC­GAG­TCC­TCT­CTCT (reverse) (NM_008509.2);* Mus musculus* peroxisome proliferator activated receptor gamma (PPAR*γ*): GAT­GGA­AGA­CCA­CTC­GCATT (forward) and AAC­CAT­TGG­GTC­AGC­TCTTG (reverse) (NM_001127330.1 and NM_011146.3; homologous to both variants). The expected product sizes were 117 bp, 80 bp, and 115 bp, respectively.

Quantitative reverse transcription-polymerase chain reactions (qRT-PCR), using both the iQ SYBR Green Supermix (Bio-Rad, USA) and the iQ5 Real-Time PCR Detection System (Bio-Rad, USA), were performed with an initial denaturation step of 3 min at 95°C followed by 40 cycles for the gene amplification step. Each cycle consisted of an initial denaturation phase of 30 s at 95°C, followed by an annealing phase of 30 s at 58°C (for LPL and PPAR*γ*) or 57°C (for Ppia), and an extension phase of 45 s at 72°C. The samples were then subjected to an extension step of 3 min at 72°C. Samples omitting reverse transcriptase were included as negative controls in each set of reactions. Ppia was amplified as a control, and its expression was used to normalize gene expression levels. Gene expression was obtained by applying the mathematical method of Pfaffl [18]. The qRT-PCR data were expressed as fold change over the control and expressed as the mean ± SEM from triplicates of the RNA samples.

### 2.5. Adipocyte Size

Samples of epididymal adipose tissue were fixed with 4% buffered formalin and embedded in paraffin. Standard sections of 5 mm were cut and stained with hematoxylin and eosin (H&E). The adipose tissue stained sections were viewed at 20x magnification, and the adipocytes area was quantified by Image J (NIH, Bethesda, MD, USA) and measuring 100 cells for each mouse (*n* = 6) and used for calculation of the average cell surface area (*μ*m^2^).

### 2.6. Hepatic Histology

Tissue samples of liver were fixed with 4% buffered formalin and embedded in paraffin. Standard 5 mm sections were cut and stained with hematoxylin and eosin (H&E), viewed with an optical microscope, and photographed at a final magnification of 100x.

### 2.7. Cell Culture and Differentiation

3T3-L1 preadipocytes cells were maintained in Dulbecco's modified Eagle's media (DMEM) that was supplemented with 10% inactivated newborn calf serum, 100 U/mL penicillin, and 0.1 *μ*g/mL streptomycin. The differentiation was initiated 2 days after confluence in DMEM containing 10% fetal bovine serum (FBS), 0.5 mM 3-isobutyl-1-methylxanthine, 0.5 *μ*M dexamethasone, and 1 *μ*g/mL insulin. After 2 days of incubation, culture medium was changed to fresh DMEM containing 10% FBS and 1 *μ*g/mL insulin. After cells were incubated for an additional 2 days, cells were continuously cultured in DMEM supplemented with 10% FBS, and medium was changed every other day for 13 days [19].

### 2.8. Oil-Red O Staining

Intracellular lipid accumulation was measured using Oil-Red O. 3T3-L1 cells were washed with phosphate-buffered saline (PBS) before being fixed for 1 h with 10% formaldehyde in PBS. The cells were stained with Oil-Red O solution (60% isopropanol and 40% water) for 20 min. The reddish dye retained by cells was eluted with isopropanol for 1 hour and the absorbance was measured using a microplate reader at 510 nm [20].

### 2.9. Western Blot Assay

Undifferentiated and differentiated 3T3-L1 cells were washed with PBS and lysed with Lysis buffer (0.5 M Tris-HCl, pH 7.4, 1.5 M NaCl, 2.5% deoxycholic acid, 10% NP-40, 10 mM EDTA). 5 *μ*g of total protein was separated on 10% SDS-PAGE gel and transferred onto nitrocellulose membrane. Incubation with primary antibodies PPAR*γ*, C/EBP*α*, C/EBP*β* and *β*-actin (1 : 1000, Santa Cruz Biotechnology) or alkaline phosphatase-conjugated secondary antibodies was overnight at 4°C or at room temperature for 1 h. Densitometric analysis of western blots was performed using ImageJ program (NIH, Bethesda, MD, USA).

### 2.10. Statistical Analysis

The results are expressed as the mean ± SEM. The IC_50_ values and their 95% confidence intervals (CI 95%) were obtained by non-linear regression. The data were analyzed by one-way analysis of variance (ANOVA) followed by Student Newman Keul's test using the GraphPad Prism 5.0 statistical analysis software (GraphPad Sotware, Inc., San Diego, CA, USA). Values of *P* < 0.05 were considered statistical significant.

## 3. Results

### 3.1. Effects of RPH and SIB Treatments on Body Weights, Net Food and Water Consumption, Relative Liver and Abdominal Adipose Tissue Weights

There were no significant differences in the initial body weights among the five groups. At the end of 15 weeks, significant differences were observed in the final body weights and the net food consumption between the control ND group (40.38 and 31.65 g, resp.) and the HFD group (55.13 and 38.24 g, resp.) (Table 1). Compared to the ND group, HFD-fed mice showed a significant increase in body weight (36.5%, *P* < 0.05), and the observed final body weights were significantly lower in animal groups that received HFD + RPH 10 mg/kg (17.0%, *P* < 0.05), HFD + RPH 20 mg/kg (%, *P* < 0.05) and HFD + SIB (28.2%, *P* < 0.05). There was a significant increase in food consumption in the HFD group (20.7%) but not in the groups treated with RPH or SIB compared to the ND group.

Net energy intake (kJ/mouse/day) was higher in the group fed the HFD than in the ND group. However, RPH (10 mg/kg) and SIB treatments significantly lowered the net energy intake. Nevertheless, no statistical difference in water consumption was observed between the groups. The relative weight of the accumulated abdominal fat was significantly higher when feeding HFD than the value for the ND mice (3.5-fold greater, *P* < 0.05), which was reduced by 2.2, 2.3 and 1.8 fold, respectively, with RPH 10 mg/kg, RPH 20 mg/kg and SIB treatments. The relative hepatic weights in the HFD group were found to be significantly higher than those of ND-fed mice. However, this increase was not seen in the animal group that received treatment with 20 mg/kg of RPH.

### 3.2. Effects of RPH and SIB Treatments on Plasma and Liver Parameters

The amylase and lipase activities were significantly higher in HFD-fed mice (64.7% and 32.4%, resp.) compared with mice fed ND. RPH 20 mg/kg and SIB treatments significantly lowered the HFD-associated increase in amylase and lipase activities (Table 2). However, RPH 10 mg/kg failed to reduce the lipase level effectively. The HFD-induced increases in plasma levels of ALT (81.4%), AST (159.9%), total cholesterol (69.6%) and triglycerides (66.7%), and in the liver levels of total cholesterol (114.6%) and triglycerides (142.2%), were also significantly decreased (*P* < 0.05) by RPH or SIB treatments (Table 2).

The plasma levels of glucose and insulin were significantly elevated in mice fed HFD (74.8% and 188.7%, resp.) compared with mice fed ND. RPH 10 and 20 mg/kg and SIB treatments caused a significant decrease of HFD-induced increases in plasma glucose levels (35.5%, 33.2% and 13.4%, resp.), and plasma insulin levels were significantly lowered by 64.8%, 52.4% and 72.7%, respectively (Table 2).

### 3.3. Effects of RPH and SIBT Treatments on Plasma Levels of Ghrelin, Leptin, Resistin, TNF-*α*, IL-6 and MCP-1

**Figure 1:**
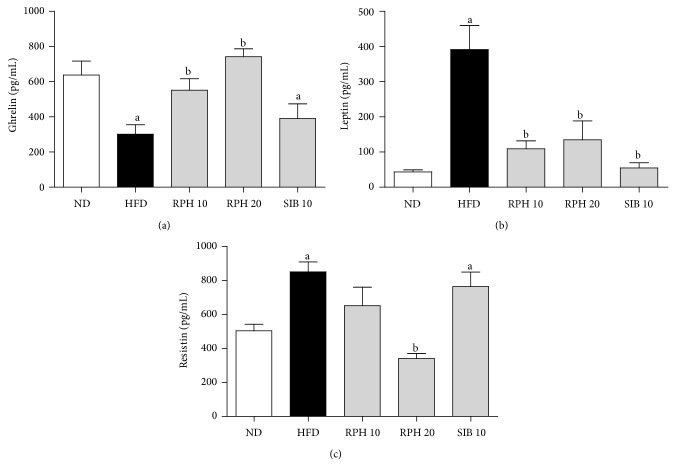
Effects of resin of* Protium heptaphyllum* (RPH) and sibutramine (SIB) treatments on plasma levels of ghrelin (a), leptin (b), and resistin (c) in mice fed experimental diets for 15 weeks. ND: normal diet; HFD: high-fat diet. Each value is the mean ± SEM (*n* = 6–8). ^a^ *P* < 0.05 versus ND group. ^b^ *P* < 0.05 versus HFD group.

**Figure 2:**
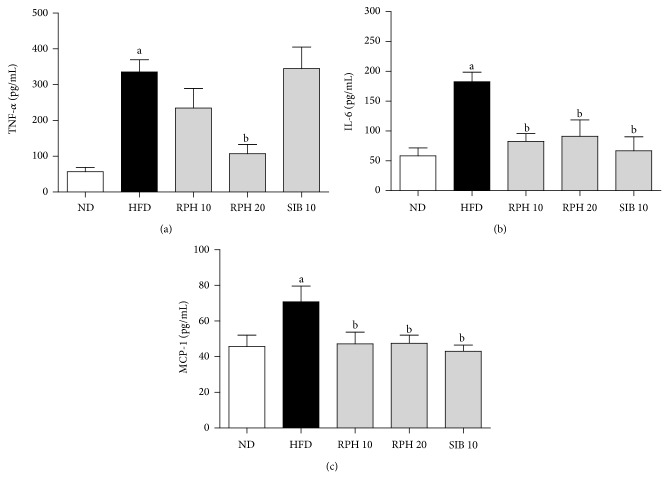
Effects of resin of* Protium heptaphyllum* (RPH) and sibutramine (SIB) treatments on TNF-*α* (a), IL-6 (b), and MCP-1 (c) levels in mice fed experimental diets for 15 weeks. ND: normal diet; HFD: high-fat diet. Each value is the mean ± SEM (*n* = 6–8). ^a^ *P* < 0.05 versus ND group. ^b^ *P* < 0.05 versus HFD group.

Mice on HFD had a lower level of ghrelin but a higher leptin and resistin levels compared to the ND group. Unlike the RPH treatment, which showed a further increase in the plasma level of ghrelin, SIB treatment had no significant influence on the HFD-induced change in ghrelin (Figure 1(a)). Plasma levels of leptin were significantly less in groups of mice treated with RPH or SIB compared to the HFD control (Figure 1(b)). While SIB treatment demonstrated no significant influence on the HFD-induced increase in the resistin level, RPH 20 mg/kg treatment significantly lowered the level of plasma resistin (Figure 1(c)). Mice on HFD demonstrated higher levels of TNF-*α*, IL-6 and MCP-1 compared to the ND group. While SIB treatment showed no significant influence on HFD-induced changes in the levels of TNF-*α*, RPH 20 mg/kg treatment caused a significant decrease in TNF-*α* level (Figure 2(a)). RPH and SIB treatments, however, reduced the IL-6 and MCP-1 levels compared to mice on HFD (Figures 2(b) and 2(c)).

### 3.4. Effects of RPH and SIB Treatments on LPL and PPAR*γ* mRNA Expression in White Adipose Tissue

**Figure 3:**
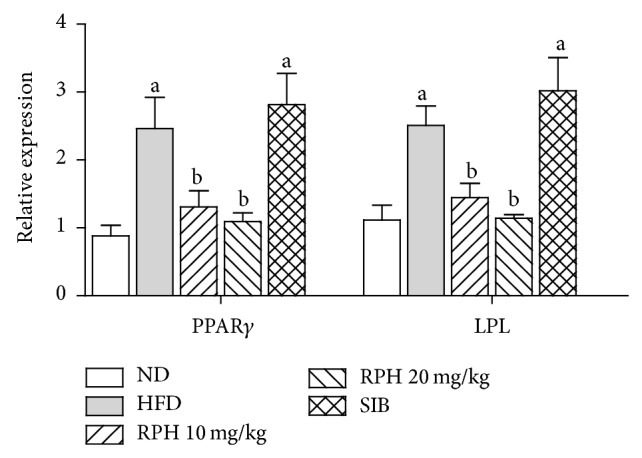
Effects of resin of* Protium heptaphyllum* (RPH) on relative gene expression of PPAR*γ* and LPL in white adipose tissue. Bars represent mean ± SEM (*n* = 6).

The expression of PPAR*γ* and LPL adipogenic genes in visceral adiposity tissue is shown in Figure 3. HFD caused significantly elevated expressions of PPAR*γ* and LPL mRNA. RPH, but not SIB treatment, resulted in significantly reduced expressions of PPAR*γ* and LPL.

### 3.5. Adipocyte Size

**Figure 4:**
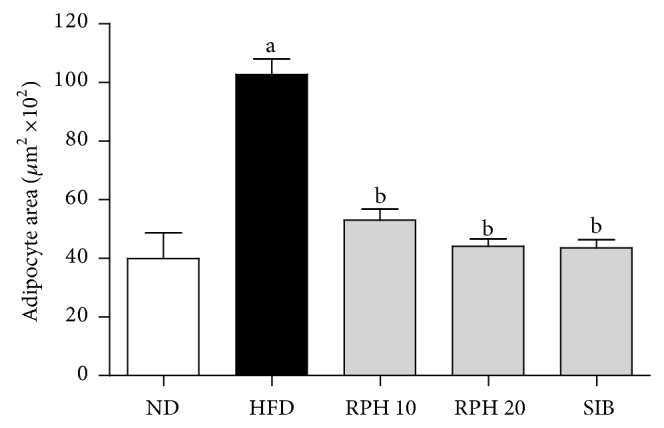
Effects of resin of* Protium heptaphyllum* (RPH) and sibutramine (SIB) treatments on adipocyte area in mice fed experimental diets for 15 weeks. ND: normal diet; HFD: high-fat diet. Each value is the mean ± SEM (*n* = 6). ^a^ *P* < 0.05 versus ND group. ^b^ *P* < 0.05 versus HFD group.

The average adipocyte size was significantly larger (*P* < 0.05) in the HFD group (101.20 ± 6.20 *μ*m^2^  × 10^2^) compared to the ND group (39.94 ± 8.85 *μ*m^2^  × 10^2^). RPH 10 and 20 mg/kg and SIB treatments significantly reduced (*P* < 0.05) the size of adipocytes by 38.3%, 48.7% and 49.4%, respectively (Figure 4).

### 3.6. Effects of RPH and SIB Treatments on Histology of Liver

**Figure 5:**
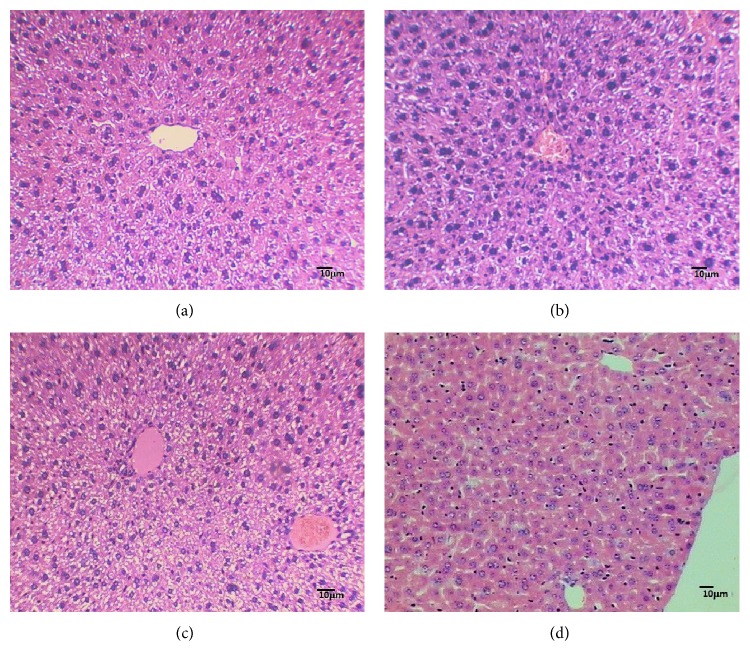
Histology of liver tissue of mice fed the experimental diets for 15 weeks. Histology of liver tissue of mice fed the experimental diets for 15 weeks. Representative microphotographs of mouse liver fed (a) a normal diet showing normal architecture and hepatocytes; (b) high-fat diet showing focal parenchymatous inflammatory infiltrate, isolated hepatocyte necrosis, and mild steatosis; (c) high-fat diet + RPH 20 mg/kg; and (d) high-fat diet + sibutramine that show well-formed nucleated hepatocytes, slight dilated sinusoids, minimal inflammatory lymphocyte infiltration, and absence of lipid droplets (H&E, ×100).

The HFD resulted in mild, focal, and parenchymatous inflammatory infiltrate, isolated hepatocyte necrosis, mild microgoticular steatosis without peculiar disposition, and the presence of lipid droplets. RPH 20 mg/kg and SIB treatments showed well-formed nucleated hepatocyte, slight dilatation of sinusoids, slight inflammatory lymphocyte infiltration, absence of fibrous tissue, and no accumulation of lipid droplets (Figure 5).

### 3.7. Effect of RPH on Lipid Accumulation and in 3T3-L1 Adipocytes

**Figure 6:**
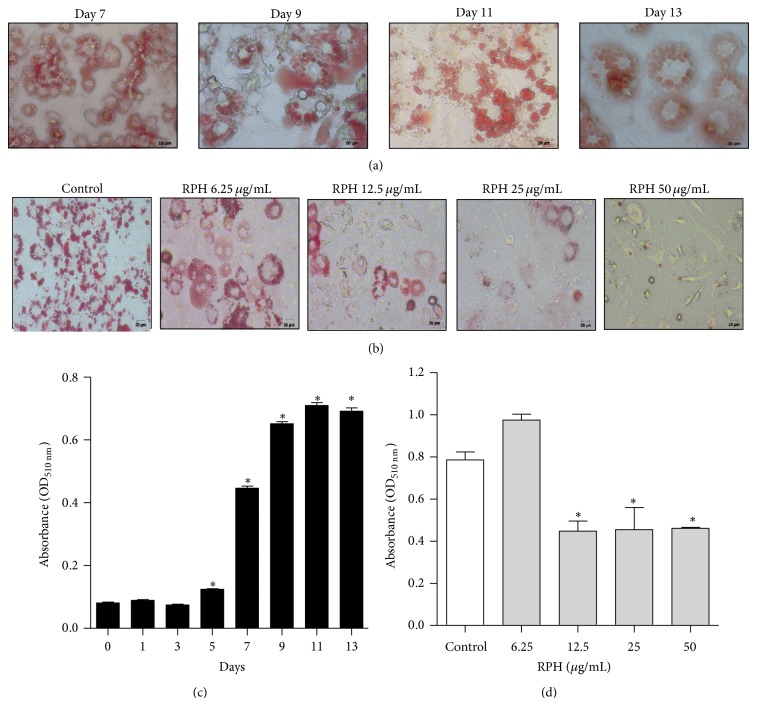
Effect of RPH on lipid accumulation in 3T3-L1 cells. Oil-Red O staining representing the effect of induction of differentiation (a) and the effect of RPH 6.25, 12.5, 25, and 50 *μ*g/mL (b) on lipid accumulation in 3T3-L1 cells (400x magnification). The staining intensity of Oil-Red O was measured at 510 nm wavelength and quantified ((c) and (d)). Values are expressed as mean ± SEM of three independent experiments. ^*^ *P* < 0.05 compared with untreated cell control (day 0).

The lipid droplets appeared at around day 5. Images of representative cells with lipid droplets on days 7, 9, 11, and 13 were shown in Figure 6(a) and the quantified staining intensity of Oil-Red O in Figure 6(c). Oil-Red O staining revealed a significant reduction in lipid accumulation with RPH (12.5, 25, and 50 *μ*g/mL) in 3T3-L1 adipocytes (Figures 6(b) and 6(d)).

### 3.8. Effect of RPH on the Protein Expression of PPAR*γ*, C/EBP*α*, and C/EBP*β* in 3T3-L1 Adipocytes

**Figure 7:**
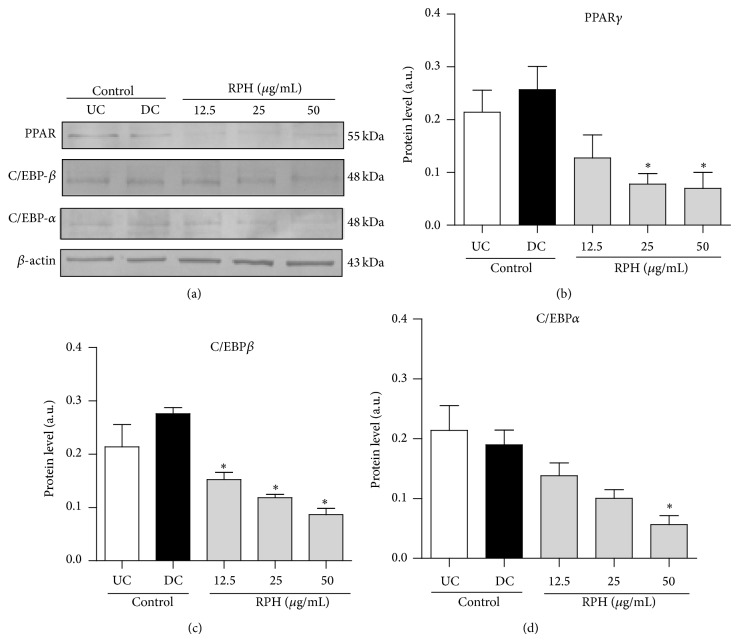
Effect of RPH on the expression of proteins PPAR*γ*, C/EBP*β*, and C/EBP*α* and in 3T3-L1 cells. *β*-actin protein was used as control. Postconfluent 3T3-L1 cells were differentiated in the absence or presence of RPH 12.5, 25, and 50 *μ*g/mL for 11 days. Proteins expression was evaluated by Western blot analysis (a). Bar graphs represent densitometric results of bands PPAR*γ* (b), C/EBP*β* (c), and C/EBP*α* (d). UC indicates undifferentiated cell control and DC indicates differentiated cell control. While RPH significantly attenuated the expression levels of C/EBP*β* at all concentration levels, statistically significant suppression was noticed for PPAR*γ* at 25 and 50 *μ*g/mL and for C/EBP*α* at 50 *μ*g/mL only. Values are shown as mean ± SEM of three independent experiments. ^*^ *P* < 0.05 compared to untreated cell control (day 0).

To investigate whether RPH (12.5, 25, and 50 *μ*g/mL) suppresses adipogenesis through a PPAR*γ* pathway, protein expressions of PPAR*γ*, C/EBP*α*, and C/EBP*β* were evaluated by Western blot analysis. The expressions of PPAR*γ*, C/EBP*α*, and C/EBP*β* were inhibited by RPH (Figures 7(a) and 7(b)). We also demonstrated that treatment with RPH resulted in suppression of PPAR*γ*, C/EBP*α*, and C/EBP*β*. While RPH significantly attenuated the expression levels of C/EBP*β* at all concentration levels, statistically significant suppression was noticed for PPAR*γ* at 25 and 50 *μ*g/mL and for C/EBP*α* at 50 *μ*g/mL only (Figures 7(c) and 7(d)). PPAR*γ* and C/EBP*α* protein levels were reduced up to 65% by treatment with 50 *μ*g/mL of RPH, while C/EBP*β* protein levels were reduced up to 50% by treatment with 50 *μ*g/mL of resin.

## 4. Discussion

Agents that can reduce body weight by exercising control at different levels such as the absorption and metabolism of carbohydrates and lipids, satiety, and lipid accumulation in adipocytes are an exciting option for the prevention and treatment of obesity. This study for the first time has assessed the effects of resin extracted from* P. heptaphyllum* (RPH) in mice fed a high-fat diet (HFD). HFD led to an increase in body weight gain, net energy intake, hyperglycemia, hyperinsulinemia, hyperlipidemia, and hyperleptinemia compared to animals fed ND. In mice fed the HFD for fifteen weeks, the administration of resin at 10 mg/kg significantly decreased the HFD-associated increase in body weight gain and net energy intake and alleviated glucose intolerance, as evidenced by decreases in plasma glucose and insulin levels. The decreased body weight gain in the RPH-treated groups of mice might be a result of slightly reduced food intake and a significant decrease in energy intake between the positive control and test groups. However, the study results show no clear dose-response effect of RPH treatment on some of the observed parameters such as body weights, net food, energy, and water intake, and relative weights of abdominal fat and liver HFD-fed mice. At times a 20 mg/kg RPH treatment mostly showed lower effects than 10 mg/kg. RPH being crude substance contains many major triterpenes and some minor chemical components, which are likely to interact changing the pharmacokinetic parameters that requires an additional study. In our previous works, resin and its major triterpenoid (*α*,*β*-amyrin) manifested a similar attitude in its gastroprotective and antinociceptive effects [9, 21].

The inhibition of digestive enzymes is one of the most widely studied mechanisms used to determine the potential efficacy of natural products as antiobesity agents [22, 23]. This study therefore addressed the effect of RPH on digestive enzymes amylase and lipase, which are secreted from the pancreas and salivary glands. Although RPH 20 mg/kg and SIB treatments significantly lowered the HFD-associated increase in amylase and lipase activities, the inhibition of amylase activity by RPH was much stronger than that of lipase.

The diet-regulating hormones ghrelin secreted from the stomach and the leptin derived from adipocytes show a major influence on energy balance by their interaction with specific receptors in the “gut-brain axis” affecting feelings of hunger and satiety [24]. Therefore, measuring their plasma levels may indicate the sensitivity of an animal to weight gain when exposed to HFD. In the present study, mice fed HFD for 15 weeks showed enhanced body weights as well as an increase in fat mass, despite an effective decrease in the circulating level of the orexigenic hormone ghrelin and an increase of the anorexigenic hormone leptin. The ghrelin and leptin secretions are possibly dysregulated with HFD, impairing homeostasis and eventually promoting obesity [25].

Interestingly, while causing no change in ghrelin, RPH treatment effectively lowered the leptin secretion, with a decrease in net energy intake. Thus, the decrease in body weight gain in HFD-fed mice by RPH treatment may be a result of reduced net energy intake in addition to the diminished fat and carbohydrate absorption. SIB, a weight control drug included in this study as a positive control, significantly blunted the abdominal body fat accumulation with a decrease in body weight and caused reductions in triglycerides and total cholesterol contents in mice fed HFD. SIB is a centrally acting serotonin/noradrenaline reuptake inhibitor that mainly increases satiety. However, SIB has now been banned due to adverse cardiovascular effects and is not recommended for hypertensive patients or in case of a history of cardio- and cerebrovascular disease [26].

Excess adipose tissue in the visceral compartment may contribute to an increase in local/systemic inflammation and enhanced insulin resistance and may even account for increased overall cardiovascular morbidity and mortality [27]. Resistin is an adipose-secreted adipokine linked to obesity and insulin resistance. Normal resistin levels are an expression of both an adequate nutritional state and controlled inflammatory state [28]. HFD-fed mice showed an increase in resistin level, which was significantly depressed by RPH treatment, possibly as a result of decreased visceral adiposity. Insulin resistance is a key factor in metabolic disorders like hyperglycemia and hyperinsulinemia, which are promoted by obesity [29].

In this study, mice on HFD demonstrated increased accumulation of abdominal fat, hyperglycemia, and hyperinsulinemia, which was significantly improved by concurrent therapy with RPH, suggesting that RPH ameliorates obesity-associated insulin resistance. Compared to animals fed ND, mice fed on HFD revealed hyperleptinemia. Although leptin is known to be a potent insulin sensitizer and can improve glucose tolerance, leptin resistance might occur in response to HFD, and both hyperleptinemia and inflammation have been proposed as causative mechanisms [24, 30].

Long-term treatment of mice with RPH for 15 weeks caused significant changes in the serum levels of lipid parameters, namely, decreased levels of total cholesterol, total triglyceride, and LDL-cholesterol, compared to HFD control mice. RPH demonstrated inhibitory activity on pancreatic lipase, so it is reasonable to assume that inhibition of digestive lipase may in part account for the decreased serum levels of lipid parameters.

Obesity and insulin resistance are characterized by hypertrophic adipocytes and a chronic low-grade inflammation in visceral fat. The latter is a result of an increased release of proinflammatory factors including interleukin-6 (IL-6), chemoattractant protein-1 (MCP-1), and tumor necrosis factor-alpha (TNF-*α*) from both adipocytes and infiltrating macrophages [31]. In this study, epididymal fat from HFD mice demonstrated elevated numbers of larger sized adipocytes in comparison with the ND mice, complying with expansion of visceral adipose tissue and hypertrophy of adipocytes. Further, these animals exhibit altered metabolism of adipose tissue, showing increased release of adipokines, IL-6, resistin and TNF-*α*, and the chemokine MCP-1, as observed in previous studies [32, 33]. Interestingly, most of these adipokines are proinflammatory mediators, which dramatically increase in the obese state and are believed to be involved in the pathogenesis of insulin resistance. Treatment with RPH has beneficial effects of attenuating the proinflammatory adipokines and improving insulin sensibility.

This study analyzed the expression levels of genes related to lipid metabolism by qRT-PCR. To examine the mechanism of lipid accumulation induced by HFD related to lipid uptake from blood into adipocytes, the mRNA level of LPL, which separates fatty acids from lipoprotein [34], was analyzed. The LPL mRNA level was significantly higher in the HFD group, whereas, in groups treated with RPH or SIB, it was downregulated. The mRNA level of PPAR*γ*, which promotes cell proliferation and is a transcription factor for many genes related to carbohydrate and lipid metabolism [35], was also significantly higher in the HFD group than in groups treated with RPH or SIB. PPAR*γ* is implicated in various metabolic disorders, including obesity, insulin resistance, and dyslipidemia. PPAR*γ* regulates fatty acid storage and glucose metabolism. The genes activated by PPAR*γ* stimulate lipid uptake and adipogenesis by fat cells. PPAR*γ* knockout mice fail to generate adipose tissue when fed a high-fat diet [36]. Moreover, drugs that target PPAR*γ* have been shown to possess anti-inflammatory effects in animal models of obesity [37]. These data indicate that RPH is potent enough to prevent lipid accumulation in adipocytes and to reverse the inflammatory state of adipose tissue, respectively, targeting LPL and PPAR*γ*.

Plant resins including RPH are generally rich in pentacyclic triterpenes, which are considered as multitarget therapeutic agents for the prevention and treatment of metabolic and vascular diseases [38] with no prominent toxicity [39]. RPH at the doses used in this study showed no adverse influence on hepatic enzymes, suggesting that it is devoid of hepatotoxicity. Besides the* in vivo* efficacy as antiobese agent in mice fed HFD, RPH* in vitro* (12.5–50 *μ*g/mL) also exhibited significant inhibitory effect on lipid accumulation in 3T3-L1 adipocytes, as measured by Oil-Red O staining. These findings indicate that RPH may be useful for the treatment of obesity and possibly reduce the risk of obesity-related diseases.

**Figure 8:**
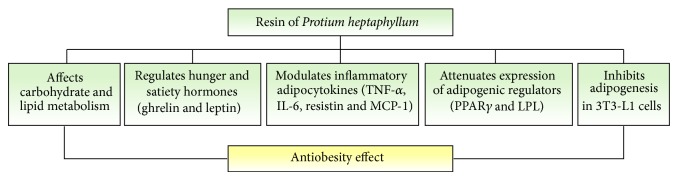
Schematic representation on the potential mechanisms for the antiobesity effect of resin* Protium heptaphyllum* (RPH) in HFD-fed mice. RPH in its antiobesity action primarily affects carbohydrate and lipid metabolism by mechanisms that include the inhibition of pancreatic enzymes (amylase and lipase), regulation of hunger and satiety hormones (ghrelin and leptin), and modulation of inflammatory adipocytokines (TNF-*α*, IL-6, resistin and chemokine MCP-1). Further it prevents adipogenesis and lipid accumulation in adipocytes attenuating the expressions of adipogenic regulators PPAR*γ* and LPL.

Finally, this study provides the scientific evidence showing that RPH has potential bioactivity for the prevention of obesity. RPH in its antiobesity action primarily affects carbohydrate and lipid metabolism by mechanisms that include the inhibition of digestive enzymes, the regulation of hunger and satiety hormones, and the modulation of inflammatory adipocytokines (Figure 8). However, future studies are needed to identify the major triterpenoid component responsible for the antiobese effects of RPH and to elucidate the underlying mechanisms.

## Figures and Tables

**Table 1:** Effects of *Protium heptaphyllum *resin (PHR) and sibutramine (SIB) treatment on body weights, net food, energy and water intake, relative weights of abdominal fat, and liver in mice fed experimental diets for 15 weeks.

Group	ND	HFD	HFD + RPH 10 mg/kg	HFD + RPH 20 mg/kg	HFD + SIB 10 mg/kg
Initial body wt (g)	25.10 ± 0.79	25.20 ± 0.55	25.20 ± 0.71	25.20 ± 0.64	25.10 ± 0.67
Final body wt (g)	40.38 ± 0.94	55.13 ± 2.35^a^	45.38 ± 1.29^b^	48.63 ± 1.47^b^	40.56 ± 1.35^b^
Net food intake (g/week)	31.65 ± 1.32	38.24 ± 3.03^a^	27.17 ± 1.17^b^	33.34 ± 1.40^b^	27.21 ± 1.16^b^
Net energy intake (kJ/mouse/day)	76.97 ± 2.89	117.06 ± 9.20^a^	83.03 ± 3.63^b^	101.86 ± 4.28	83.25 ± 3.63^b^
Net water intake (mL/week)	48.13 ± 0.73	47.63 ± 1.66	44.44 ± 1.20	41.91 ± 1.38	42.54 ± 0.72
Abdominal fat (mg/10 g of body wt)	195.4 ± 24.59	695.4 ± 96.60^a^	421.5 ± 79.22^b^	457.6 ± 28.38^b^	359.1 ± 62.21^b^
Liver wt (mg/10 g of body wt)	337.9 ± 13.60	413.1 ± 7.32^a^	378.5 ± 16.69	341.5 ± 9.56^b^	368.4 ± 11.54

Values are mean ± SEM (*n* = 8). ND: normal diet; HFD: high fat diet; RPH: resin of *Protium heptaphyllum*; SIB: sibutramine. ^a^ *P* < 0.05 versus mice fed ND; ^b^ *P* < 0.05 versus mice fed HFD (ANOVA followed by Student-Newman-Keuls test).

**Table 2:** Effects of *Protium heptaphyllum *resin (PHR) and sibutramine (SIB) treatment on serum and liver parameters in mice fed experimental diets for 15 weeks.

Group	ND	HFD	HFD + RPH 10 mg/kg	HFD + RPH 20 mg/kg	HFD + SIB 10 mg/kg
Amylase (U/L)	99.73 ± 4.14	164.30 ± 11.46^a^	123.20 ± 12.73^b^	109.90 ± 9.59^b^	101.00 ± 8.74^b^
Lipase (U/L)	223.30 ± 10.27	295.70 ± 25.62^a^	294.30 ± 12.51	221.10 ± 9.78^b^	251.30 ± 9.53^b^
ALT (U/L)	41.75 ± 3.06	75.75 ± 10.99^a^	35.13 ± 2.63^b^	40.63 ± 8.92^b^	48.38 ± 5.48^b^
AST (U/L)	72.71 ± 10.55	189.0 ± 10.24^a^	69.63 ± 5.21^b^	64.63 ± 3.22^b^	71.50 ± 8.18^b^
Glucose (mg/dL)	125.30 ± 8.34	219.10 ± 15.68^a^	142.30 ± 11.12^b^	146.40 ± 7.16^b^	177.30 ± 11.67^b^
Insulin (ng/mL)	1.059 ± 0.14	4.524 ± 0.58^a^	1.408 ± 0.11^b^	1.489 ± 0.27^b^	0.8588 ± 0.08^b^
Total cholesterol (mg/dL)	121.80 ± 3.82	206.60 ± 13.31^a^	171.00 ± 6.49^b^	151.20 ± 10.37^b^	171.40 ± 6.28^b^
Triglycerides (mg/dL)	110.20 ± 11.11	183.40 ± 13.78^a^	84.20 ± 6.19^b^	94.20 ± 6.61^b^	106.00 ± 6.88^b^
Liver total cholesterol (mg/g)	6.29 ± 0.92	13.50 ± 1.07^a^	7.57 ± 1.36^b^	7.43 ± 0.97^b^	5.43 ± 0.29^b^
Liver triglycerides (mg/g)	53.38 ± 8.29	129.30 ± 18.23^a^	74.29 ± 14.45^b^	70.63 ± 10.22^b^	47.75 ± 6.25^b^

Values are mean ± SEM (*n* = 8). ND: normal diet; HFD: high fat diet; RPH: resin of *Protium heptaphyllum*; SIB: sibutramine. ^a^ *P* < 0.05 versus mice fed ND; ^b^ *P* < 0.05 versus mice fed HFD (ANOVA followed by Student-Newman-Keuls test).
